# Intracranial Hemorrhage following Spinal Surgery: A Systematic Review of a Rare Complication

**DOI:** 10.1055/s-0042-1743525

**Published:** 2022-03-03

**Authors:** Tariq Al-Saadi, Yahya Al-Kindi, Moosa Allawati, Hatem Al-Saadi

**Affiliations:** 1 Department of Neurology and Neurosurgery, Montreal Neurological Institute and Hospital- McGill University, Montreal, Canada; 2 Neurosurgery Department, Khoula Hospital, Muscat, Sultanate of Oman; 3 Sultan Qaboos University, College of Medicine and Health Sciences, Muscat, Sultanate of Oman; 4 Department of General Surgery, Sohar Hospital, Sohar, Sultanate of Oman

**Keywords:** spinal surgery, intracranial hemorrhage, hematoma, hemorrhage

## Abstract

**Introduction** Intracranial hemorrhage (ICH) is a potentially severe complication of spinal surgeries. The occurrence of such complications causes deterioration of the patient's clinical status and delayed discharge from the hospital. Although no specific etiological factors were identified for this complication, but multiple risk factors might play role in its development, they include the use of anticoagulants, presence of uncontrolled hypertension, and perioperative patient positioning.

**Aim** A systematic review of the literature to investigate the prevalence of different types of intracranial hemorrhages in patients who underwent spinal surgeries.

**Methods** A literature review was conducted using multiple research databases. Data were extracted using multiple variables that were formulated incongruent with the study aim and then further analyzed.

**Results** A total of 79 studies were included in our analysis after applying the exclusion criteria and removing of repeated studies, 109 patients were identified where they were diagnosed with intracranial hemorrhage after spine surgery with a mean age of 54 years. The most common type of hemorrhage was cerebellar hemorrhage (56.0%) followed by SDH and intraparenchymal hemorrhage; 23.9 and 17.4%, respectively. The most common spine surgery was laminectomy (70.6%), followed by fixation and fusion (50.5%), excision of spinal lesions was done in 20.2% of the patient, and discectomy (14.7%).

**Conclusion** The data in this study showed that out of 112 patients with ICH, cerebellar hemorrhage was the most common type. ICH post–spine surgery is a rare complication and the real etiologies behind this complication are still unknown, cerebrospinal fluid drain and durotomy were suggested.

Intracranial hemorrhage (ICH) is a rare but a potentially severe complication of spine surgery that can lead to prolonged hospital stay, persistent neurological impairment, and even death. 1 It is a devastating subtype of hemorrhagic stroke with mortality up to 50% by 1 month where it can also cause permanent neurological disabilities and increase the need for long-term rehabilitation. 2

Common etiologies of postoperative intracranial hemorrhage may include the use of anticoagulants, blood clotting dysfunction, incomplete hemostasis of the dura mater or diploe, poorly controlled high arterial blood pressure, and the misuse of rigid pins for head fixation. Deterioration during the postoperative period should alert physicians to the possibility of intracranial hemorrhage. Emergent neuroimaging should be performed to exclude the diagnosis and facilitate rapid intervention. 3

The first case reported concerning ICH after spinal surgery was in 1981 by William M. Chadduck and many cases have been reported since that time. 1 There has been an increased interest in ICH after spine surgeries in the past 10 years, but no specific etiological factors have been identified yet for this type of complication. A retrospective study from 2007 to 2012 identified 371 of 167,106 (0.22%) patients who developed stroke after spinal surgery, 53 of those patients (14.2%) developed hemorrhagic stroke. 4 Moreover, cerebral hemorrhage accounts for the vast majority of postoperative ICH, subdural hemorrhage/hematoma (SDH), subarachnoid hemorrhage (SAH), and intraventricular hemorrhage/hematoma (IVH) which are other forms of postoperative hemorrhages. Depending on the location of the hemorrhage, patients may present with headache, altered level of consciousness, nausea, vomiting, and/or dysarthria. 5 Due to the extremely low frequency of occurrence, there is still no satisfactory explanation for the mechanism of this unpredictable complication. ICH as a result of cerebral sag also has been postulated; cerebral sag resulting from intraoperative or postoperative active cerebrospinal fluid (CSF) drainage might stretch and occlude the bridging cerebellar veins leading to hemorrhagic venous infarction. 1 2

Perioperative patient positioning is another factor that is widely suspected to contribute to remote cerebellar hemorrhages (RCH) but the relevance of this is unclear. 2 In 2006, a literature review showed that 10 cases of RCH which had been reported to date, and they found no correlation between RCH and age, sex, pathology operated, and type of interventions performed. 6 A recent systematic review published in 2016 included 44 articles (57 patients with RCH) and aimed to identify the link between procedures and the occurrence of RCH and its possible risk factors; they found that RCH was most frequently reported as a consequence of decompressive procedures used to treat spinal stenosis and spinal canal stenosis. 7

Spontaneous intracranial epidural hematoma (EDH) as a complication of spinal surgery is extremely unusual. 8 Depending on the patient's condition and neurological status, the treatment can be conservative or surgical by evacuation or decompressive craniotomy and drainage. 9

The aim of this systematic review is to identify the causes and risk factors of intracranial hemorrhages after spinal surgeries and how can they be prevented aiming to decrease the mortality and the hospital stay for those who recently underwent spine surgery.

## Methodology

### Literature Search and Formulating Selection Criteria

This study is a systematic review that is aiming to study the prevalence of different types of intracranial hemorrhages postspinal surgeries. The following search engines were used: a bibliographic electronic database, EBSCO, Proquest, Wiley, SpringerLink, Google Scholar, SAGE Journals, ScienceDirect, and BioMed Central looking for the related studies. Date of searching were started on first of May to the end June, data were collected irrespective to the publication date.

The keywords used were “Intracranial haemorrhage,” “Intracranial hematoma,” “Intracerebral haemorrhage,” “cerebellar haemorrhage,” “subdural hematoma,” “epidural hematoma,” “laminectomy,” “spinal fusion,” “screw fixation,” “Harrington rod placement,” “discectomy,” and “tumour resection.”

### Inclusion Criteria

Furthermore, studies were included if they met the following inclusion criteria: (1) were case report or case series, (2) case report of ICH after spine surgery, and (3) were published in English language. There was no age restriction and articles from around the world were searched.

### Exclusion Criteria

Excluded articles were articles that involved the occurrence of only spinal hemorrhage after spinal surgery and studies that involved intracranial hemorrhage after spinal procedures that did not involve surgical interventions (including lumbar puncture and nerve block procedures or spinal anesthesia). Other exclusion criteria extending to studies that were unavailable in English, noncompleted, repeated, or did not meet any of the previously mentioned criteria.

### Data Extraction

Two independent reviewers evaluated the titles and abstracts according to the inclusion and exclusion criteria. For each potentially eligible study, two reviewers assessed the full text. After looking into the details of each study, data were extracted using multiple variables that were consistent with the study aim. The variables included article type, author's name, the year the study was published in, and sample size of each study.

Demographic data of the patients were collected, and it included the age, gender, and presence of comorbid conditions such as diabetes mellitus. Presence of previous intracranial surgery in the past surgical history was obtained too. The details of the spine surgeries, the patients went through were obtained and they included the level of spine surgery, indication for spine surgery, type of spine surgery, volume of CSF drain in milliliters, presence of dural opening, drain placement, and presence of CSF leak. Details of each type of hemorrhage that occurred were also taken in details including the duration till the onset of symptoms, type of hemorrhage, type of intervention, and outcome after treating the hemorrhage. All of the collected data are listed in ( Supplementary S1 ; available in the online version).

Regarding the systematic review articles that reviewed articles in their discussion section, the studies mentioned in those review articles were reviewed by the authors and the same data were extracted above.

**Fig. 1:**
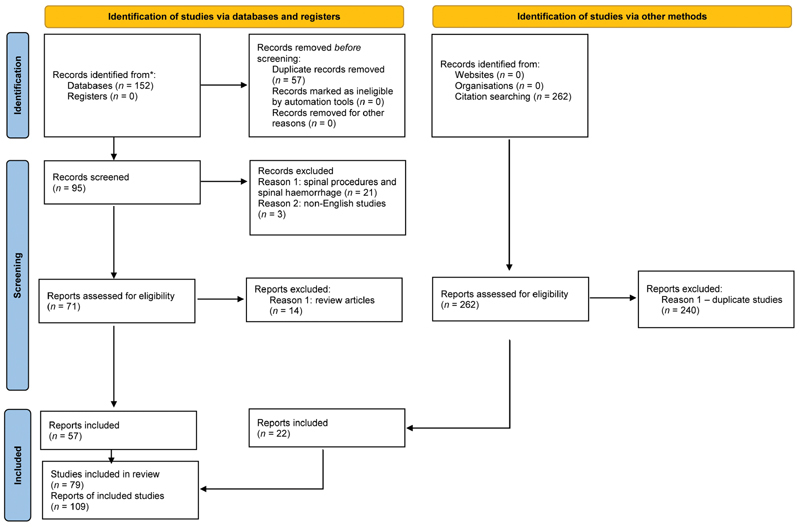
PRISMA flow diagram. PRISMA, Preferred Reporting Items for Systematic Reviews and Meta-analyses.

The Preferred Reporting Items for Systematic Reviews and Meta-analyses (PRISMA) statement was used to guide the conduction and reporting of this review. Fig. 1 represents a flow chart depicting the study selection process.

A total of 152 studies were obtained through database searching and 0 studies were obtained from registries. A total of 95 articles remained after eliminating the duplicates manually by two different reviewers and a word document was used to list all the article titles including authors name and year of publication. A total of 21 articles were eliminated as they were related to the occurrence of hemorrhage after spinal procedures that did not include surgical interventions, such as lumbar puncture and spinal anesthesia; furthermore, they were related to the occurrence of spinal hemorrhage after spinal surgeries and we removed. As studies were screened, three non-English studies were excluded. Out of 71 studies that were assessed for eligibility, 14 studies were eliminated as they were review articles; however, those review articles were used to get further case reports and case series to analyze them. Finally, 57 relevant studies after screening and assessing studies were got from databases.

Regarding the review articles that were found, they were used to get 262 relevant studies, and all author names and years of publication were noted down. Out of those studies, 240 studies were eliminated due to repetition/duplications. Finally, 22 articles from other methods were obtained, a total of 79 studies and 109 cases that were reported and statistically analyzed.

### Data Analysis

After noting down the data in an excel sheet, data were analyzed using statistical methods through the SPSS software. Using the software, discrete data were investigated through frequency tables to find the percentages of patients who had specific spinal diseases, the types of surgeries they underwent, and the types of hemorrhage they developed postoperatively.

The relations between variables were investigated using cross-tabulations and Chi-square method to find if there were any associations between many variables such as age on one hand and the types of hemorrhage on the other hand and whether it was a positive or a negative association (clearly identified by the *p* -value). Other investigated associations included the relation between CSF leak and number of spine surgeries each patient underwent. Regarding the data that is not related to the surgeries themselves, comorbid conditions that the patients suffered from, like diabetes mellitus (DM) and/or arterial hypertension (AH), were identified and analyzed.

All the collected data were presented in the forms of graphical representations such as bar charts where different categorized variables were portrayed like age and gender plus their relations.

### Methodological Quality

To ensure that case reports and case series that were included in this review, a critical appraisal tool created by an Australian institute called the Joanna Briggs Institute was followed. 10 Regarding the case reports, basic demographic information, such as age and gender, was mentioned, the patients' history was described in a detailed way as a timeline to display the progression of the symptoms along with the current clinical condition. The diagnostic tests such as brain imaging modalities and the types of treatment (whether it was conservative or surgical) were mentioned along with postintervention updates of the patients' conditions and outcomes. The adverse and unanticipated events of the surgery were included.

Regarding the case series, clear inclusion criteria to include specific patients were explained, the case series had complete inclusion of the patients. All the patients had valid diagnostic methods such as brain imaging done on them to diagnose the postoperative complication evaluated in this study. Basic demographic information and clinical information along with the outcome after the surgery/ treatment were explained. There was a clear reporting of the presenting sites (hospitals) where the patients were admitted and asked for clinical information. Statistical analysis was appropriate and sufficient to take lessons from it.

## Results

A total of 109 cases have been identified; female patients constituted 60.6% of all patients while male patients were 39.4%. The mean age of patients in the studies was between 54 and 55 years. Patients were further classified to three age groups to allow comparison between them. Patients in middle age group, 15 to 65 years, accounted for the majority of patients (62.4%), while patients in the youngest age group (<15) and the oldest age group (>65) comprised 7.3 and 30.3% of the whole study group, respectively.

The most common comorbid condition recorded in this review was AH (8.0%) followed by other comorbidities (8.0%) such as end-stage renal disease, previous stroke, Crohn's disease, Factor V Leiden, coronary artery disease, and other comorbidities. DM was only present in 0.9% of the cases. Moreover, the presence of both DM and AH in the cases was recorded as 5.4%.

Table 1 displays the characteristics of the spine surgeries done in this study group. Majority of the cases in our research have done more than one type of intervention; however, this table represents the prevalence of each intervention. The most commonly performed spine intervention was decompressive laminectomy which was done in 70.6% of the cases, followed by fusion and fixation which were performed in 50.5% of the cases. Resection of lesions and discectomy were performed in 20.2 and 14.7% of the patients, respectively. Minority of the cases (5.5%) underwent vertebroplasty or kyphoplasty. The indications of those types of spine surgeries were not similar among the patients as 26.6% of the patients were diagnosed with spinal stenosis, followed by a spine tumor in 20.2% of the patients. Developmental anomalies were present among 19.3% of the patients while a herniated disc was present in only 12.8% of the patients. Moreover, 19.3% were having age-related degenerative condition.

**Table 1:** Characteristics of spine surgeries

Previous intracranial surgery *n* (%)	1 (0.9%)	
Spine surgery types	Decompressive laminectomy	80 (71.4%)
	Discectomy	18 (16.1%)
	Resection/excision of spinal lesions	23 (20.5%)
	Vertebroplasty/kyphoplasty	6 (5.4%)
	Fusion and fixation	57 (50.9%)
Levels of spine surgery	Cervical	17 (15.2%)
	Thoracic	14 (12.5%)
	Lumbar	75 (67.0%)
	Sacral	6 (5.4%)
Dural opening	87 (77.7%)	
Postoperative drain placement	71 (63.4%)	
CSF volume (mL)	Range	100–1,300
	Mean ± SD	525 ± 295

Abbreviations: CSF, cerebrospinal fluid; SD, standard deviation.

In this study, the levels of spine surgeries were identified and subclassified into four groups as cervical, thoracic, lumbar, and sacral. Spinal surgeries in the lumbar region were done in 67.0% of the cases, while operations in the cervical and thoracic regions were done in 15.6 and 12.8% of the cases, respectively, and only 2.8% of the cases involved a sacral surgery; however, 16 cases were not reported. Furthermore, majority of the surgeries (77.1%) involved making a dural opening whether it was done accidentally or intentionally, and 62.4% of the patients had a drain placed after the surgery. In addition, the mean CSF volume drained and/or leaked was 521 mL.

### Prevalence of Different Types of Intracranial Hemorrhages/Hematomas

**Fig. 2:**
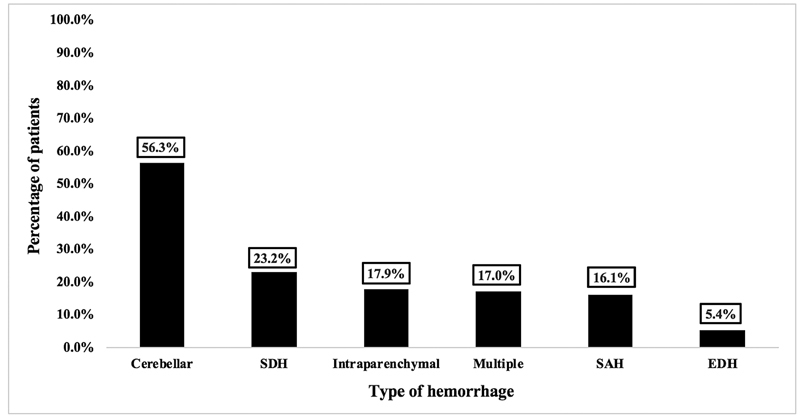
Prevalence of the types of hemorrhage. EDH, epidural hematoma; SAH, subarachnoid hemorrhage; SDH, subdural hematoma.

Fig. 2 illustrates common types of hemorrhages/hematomas that developed in patients after spinal surgeries. The most common type of hemorrhage seen in those patients was cerebellar hemorrhage (56.0%) and the least common type of hemorrhage was epidural hematoma (5.5%). SDH and intraparenchymal hemorrhage were seen in 23.9 and 17.4% of the cases, respectively. Patients who developed multiple types of hemorrhage, such as a combination of SDH and SAH, were seen in 19 (17.4%) of the patients.

### Locations of Different Types of Intracranial Hemorrhages/Hematomas

**Fig. 3:**
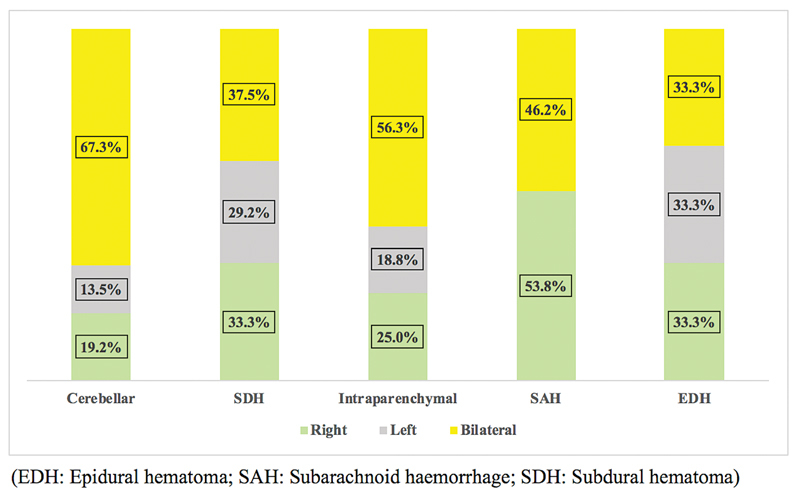
Anatomical locations of intracranial hemorrhages. EDH, epidural hematoma; SAH, subarachnoid hemorrhage; SDH, subdural hematoma.

Fig. 3 illustrates the anatomical locations where each type of hemorrhage was seen the most. Concerning cerebellar hemorrhage, 66.0% of those cases involved bilateral hemorrhage, followed by bilateral intraparenchymal hemorrhage which occurred in 56.3% of the cases. Hemorrhage in the left side of the brain was the least common among all reviewed cases. Percentage of patients with right-sided hemorrhage was the highest in cases of SAH (53.8%) and patients with left sided SAH were not identified.

### Prevalence of Each Type of Hemorrhage According to Different Variables

Table 2 showed the prevalence of each type of hemorrhage. Patients in middle age group, 15 to 65 years, accounted for the majority of patients, and 65.1% of them developed significant cerebellar hemorrhage ( *p* = 0.004). Other significant findings in the middle age group included the occurrence of SDH in 53.8% of them ( *p* = 0.024) and occurrence of EDH in 66.7% of them ( *p* = 0.019), while patients in the youngest age group (<15) did not develop cerebellar hemorrhage. In addition, the occurrence of SAH after surgery was significantly seen more in the oldest age group (>65) compared with the other age groups (55.6%; *p* = 0.029).

**Table 2:** Comparison between different variables and types of hemorrhage

Variable	Classification	Types of hemorrhage *n* (%)					Total *n* (%)
		SDH	EDH	SAH	Intraparenchymal	Cerebellar	
Age, median (range)		46.7 (6–82)	31.5 (14–58)	63.1 (10–77)	54.0 (23–75)	58.8 (23–99)	–
Levels of spine surgery ( *n* )	Cervical	4 (15.4)	1 (16.7)	4 (22.2)	1 (5.3)	13 (20.6)	17 (100.0)
	Thoracic	2 (7.6)	0 (0.0)	1 (27.8)	6 (30.0)	8 (12.7)	14 (100.0)
	Lumbar	18 (69.2)	5 (83.3)	12 (66.7)	11 (55.0) **a**	38 (60.3)	73 (100.0)
	Sacral	2 (7.6)	0 (0.0)	1 (5.5)	1 (5.3)	0 (0.0)	3 (100.0)
Age	<15	5 (19.2)	2 (33.3)	1 (5.5)	0 (0.0)	0 (0.0)	8 (100.0)
	15–65	14 (53.8) **a**	4 (66.7) **a**	7 (38.9)	15 (78.9)	41 (65.1) a	68 (100.0)
	>65	7 (26.9)	0 (0.0)	10 (55.6) **a**	4 (21.1)	20 (31.7)	33 (100.0)
Dural opening ( *n* )	–	18 (69.2)	6 (100)	12 (66.7)	17 (89.5)	47 (74.6)	84 (100.0)
Postoperative drain, n	–	12 (46.1)	4 (66.7)	12 (66.7)	12 (63.2)	40 (63.5)	68 (100.0)
Treatment	Conservative	13 (50.0)	0 (0.0)	9 (50.0)	13 (68.4)	32 (50.8)	56 (100.0)
	Surgical	11 (42.3)	6 (100) **a**	9 (50.0)	6 (31.6)	27 (42.9)	49 (100.0)
Reported CSF leak	–	5 (19.2)	1 (16.7)	2 (11.1)	4 (21.1)	16 (25.4)	26 (100.0)
Death	–	0 (0.0%)	0 (0.0)	2 (11.1)	3 (15.8)	1 (1.6)	4 (100.0)
Onset of symptoms	Less than 24 hours	9 (34.6)	4 (66.7)	9 (50.0)	6 (31.6)	16 (25.4)	35 (100.0)
	After 24 hours	15 (57.7)	2 (33.3)	9 (50.0)	12 (63.2)	39 (61.9)	66 (100.0)
Total		26 (100.0)	6 (100.0)	18 (100.0)	19 (100.0)	63 (100.0)	109 (100.0)

Abbreviations: CSF, cerebrospinal fluid; EDH, epidural hematoma; SAH, subarachnoid hemorrhage; SDH, subdural hematoma.

a Null hypothesis is rejected as *p* -value is below 0.05

A total of 60.3% of patients who underwent a lumbar surgery, which is the most common operated region, developed cerebellar hemorrhage. Out of the 19 patients who suffered from an intraparenchymal hemorrhage, 30.0% of them had a surgery in the thoracic segments, and 55.0% of them significantly had underwent surgeries in the lumbar region ( *p* = 0.028).

Other findings in this study showed that only one patient had a previous intracranial surgery. Forty-seven out of 63 cerebellar hemorrhage cases had dural opening. The placement of postoperative drains in the patients predisposed to the development of cerebellar hemorrhage in 63.5% of the patients. Regarding the modality of treatment used to treat the hematoma/hemorrhage, occurrence of EDH after spinal surgery was more likely to be treated surgically than with conservative treatment (100.0%; *p* = 0.011).

Generally, out of all types of hemorrhage that were treated surgically, the most common type was cerebellar hemorrhage (42.9%) followed by SDH (42.3%). Different types of hemorrhage/hematoma were treated with different types of surgeries where out of 109 cases, 19 patients (17.0%) were treated using external ventricular drain (EVD) insertion, 14 (12.5%) patients were treated with craniotomy, 6 (5.4%) patients with craniectomy, and 4 (3.6%) patients with burr hole.

Finally, regarding the presence of CSF leak after the surgery, 25.4% of cerebellar hemorrhage cases had the leak. Moreover, the highest rate of death was seen among intraparenchymal hemorrhage patients (15.8%). Out of all cerebellar hemorrhage cases, 25.4% presented within 24 hours, while a greater percentage of almost 62.0% presented after 24 hours.

### Number of Hemorrhage Types According to Different Variables

Table 3 illustrates the comparison between different variables and whether the patient had one type of hemorrhage or more than one type. The 56 patients (68.3%) who presented to the hospital after 24 hours were likely to be suffering from one type of hemorrhage than more than one type. There was no significant difference in the number of types of hemorrhage among patients who died. And patients who did not develop postoperative complications (e.g., CSF leak, seizure, hydrocephalus, and others) were significantly more likely to be suffering from one type of hemorrhage ( *p* = 0.034).

**Table 3:** Comparison between different variables and number of hemorrhage types

Variable		One type of hemorrhage *n* (%)	More than one types of hemorrhage *n* (%)	Total *n* (%)	*p* -Value
Onset	Less than 24 hours	26 (31.7)	10 (52.6)	36 (100.0)	>0.05
	More than 24 hours	56 (68.3)	9 (31.7)	65 (100.0)	
Death	No death	88 (97.7)	17 (89.4)	105 (100.0)	>0.05
	Death	2 (2.2)	2 (10.5)	4 (100.0)	
Complications	No complications	30 (33.7)	12 (63.2)	42 (100.0)	0.034
	Complications	59 (66.3)	7 (36.8)	66 (100.0)	

## Discussion

ICH is a rare but a potentially severe complication of spine surgery that can lead to persistent neurological impairment, and even death. 1 This literature review was conducted and included a total of 109 cases that involved the development of intracranial hemorrhage after a spine surgery. Mean age of all 109 patients was 54.5 ± 18.8 years and the median was 57 years. Comparing this to a case series of eight patients published in 2013, the median age among the eight-case series was 63.5 years. 11 This review found that the prevalence of ICH post–spine surgery was higher in the middle age group (15–65 years) with a percentage of 62.4%, followed by the age group of more than 60 and less than 15 years with percentages of 30.2 and 7.3%, respectively. The prevalence of intracranial hemorrhage as a complication of spine surgery in this study was higher in females (60.6%) compared with male patients (39.4%). In comparison to a systematic review done to identify the impact of sex in the development of postoperative complications of spine surgeries which concluded that the odds of mortality were higher in males but not of complications after spine surgery. 12 However, another study was done to identify the possible risk factors of postoperative spinal epidural hematoma after lumbar decompression which showed that females have higher risk (64%) compared to males (36%). 13 In this article, prevalence of AH alone was 8.0% and the prevalence of DM alone was 0.9%, prevalence of DM and HTN combined was 5.4%, while the prevalence of other comorbidities was 8.0%.

Regarding the types of the spine surgeries, this study found that decompressive laminectomy was the most commonly performed surgery done in 70.6% of the cases followed by resection of spine lesions which was done in 20.2% of the cases. In comparison to a prospective study to identify early complications related to spine surgery, fusion was the most common performed surgery followed by decompressive laminectomy. 14

In this article, surgeries in the lumbar region were done in 67.0% of the cases, while operations in the cervical and thoracic regions were done in 15.6 and 12.8% of the cases, respectively, and only 2.8% of the cases involved a sacral surgery. Comparing those findings to a study published in 2016 about the same topic, Pham et al found that out of 1,113 patients who underwent spinal surgeries, 58% of the surgeries involved the lumbar/sacral regions, 29% involved the cervical region, 7% in the thoracolumbar region, 4% in the thoracic region, and 2% in the cervicothoracic region. 15 However, it is difficult to establish a relationship between the location of spine surgery and the incidence of ICH, as this study is a systematic review.

Dural tear is a well-known complication of spine surgery. This review found that the dural tear was reported in 77.1% of the cases. Comparing this finding to another study reported the incidence to be 3.5 to 17.4%, and it is associated with many different conditions such as pseudo meningocele, CSF fistula formation, meningitis, nerve root or brain stem herniation, cerebellar dysfunction, and intracranial hemorrhage in different location. 16 Furthermore, this study found that the postoperative drains were inserted in 62.4% of the patients. This is comparable to a case series of eight patients who had a subfascial or subcutaneous postoperative drain placed. This was justified by stating that drains are an important part in the postoperative care of many neurosurgical patients even if an intraoperative CSF leak was absent. 11 Regarding the CSF leak after spinal surgeries, a study published in 2019 concluded that the overall incidence of CSF leak after spinal surgeries involving resection of both intradural and extradural pathologies is relatively low (6.6%). 17 According to this review, we found that the volume ranged from 100 18 19 to 1,300 mL 20 with a mean volume of 521 mL. Another review showed that the mean volume of CSF drained, prior to the appearance of the symptoms of ICH, among 8 patients was 225 mL. 11

The incidence of ICH post–spinal surgery is very rare. A total of 1,113 cases were reviewed retrospectively from 2008 to 2013 in a study done in a medical center in the United States, out of all cases, six (0.4%) were found to have subdural hygromas suggestive of acute intracranial hypotension, remote cerebellar hemorrhage, or subdural hematoma. 15 This review identified that there are five distinct forms of hemorrhages with varying incidence of occurrence associated with spinal surgery and they have been classified according to their locations: cerebellar hemorrhage (56.0%), SDH (23.9%), intraparenchymal hemorrhage (17.4%), SAH (16.5%), and EDH (5.5%). However, 17.4% of the cases had more than one type of hemorrhage.

Cerebellar hemorrhage accounts for 9 to 10% of all ICH. 21 The occurrence of cerebellar hemorrhage after spine surgery is considered a rare complication with reported incidence of 0.08%. This review identified 61 out of 109 cases who developed this type of hemorrhage and accounted for the majority of hemorrhages in this review. In addition, another study found that cerebellar hemorrhage was higher in middle-aged and older adults which is similar to what this review found, as (68) 62.4% of those aged from 15 to 60 years ( *p* = 0.004), and in 41 cases, age was more than 65 years in developed cerebellar hemorrhage post–spine surgery. Moreover, patients in the youngest age group (<15 years) did not develop cerebellar hemorrhage. Cerebellar hemorrhage can occur after any type of spine surgery 22 ; however there is no agreement on exact pathology that causes it; however, majority of authors agree that cerebellar hemorrhage is venous in origin and results from CSF leak or dural opening before or after the surgery. 2 In addition, they support the fact that cerebellar hemorrhage mostly occurs bilaterally in the sites where the cerebellar draining veins are located. 23 This is also the finding of this study where 66.0% of the cases of cerebellar hemorrhage were bilateral. One case report was studying the real cause of cerebellar hemorrhage after spine surgery where an operation was done to treat the hemorrhage and they took a biopsy to know the underlying pathological cause, which showed that the patient also had arteriovenous malformations (AVM), and authors concluded that it is difficult to know the underlying pathological cause, as this patient was having CSF leak also during spine surgery. 24 This may raise questions concerning the link between CSF leak and dural opening with cerebellar hemorrhage, However, in this review, 74.6% who developed cerebellar hemorrhage had a dural opening done intentionally or accidently, and drain were placed in 63.5% of all cerebellar hemorrhage cases and 25.4% of cerebellar hemorrhage patients were reported CSF leak which was the highest among all the type of ICH. These three high percentages could support what was already hypothesized by most of the authors; however, it is not definitive because the most common type of ICH in this review was cerebellar hemorrhage.

In this review, as it shown in Table 2 , 74.6% of patients who developed cerebellar hemorrhage had an accidental or intentional dural opening. This may also support the hypothesis of the relation between dural opening and cerebellar hemorrhage. This type of hemorrhage could also be explained by large CSF drainage, or CSF leak may result in downward cerebellar displacement, or “sag,” resulting in stretch occlusion of superior cerebellar veins draining in the cephalad direction into the deep venous system. This may cause intracerebellar hemorrhage in patients with insufficient venous collaterals. 21 However, authors also suggested that because of high incidence of dural opening and high prevalence of spine surgery, the number of patients who developed cerebellar hemorrhage could be also higher because most of the cerebellar hemorrhage cases consist of small bleeding which have an asymptomatic course and are not detected by the CT scan. 22

This study found that out of 109 cases, EDH was present only in 5.5% of cases. A previous study mentioned that EDH is extremely rare after spinal surgery, 25 but despite its rarity, this uncommon complication may result in devastating neurological sequelae, including lower limb weakness. 13 Unlike cerebellar hemorrhage and SDH which have a venous origin, SDH is caused by the dissecting force of the dura-cranium adhesion, and it is well known that the adhesion between the dura mater and the cranial vault bone is less tight in the elderly. 8 This hypothesis is supported by this study as none of the patients in the oldest age group (>65 years) developed EDH, and it was more seen in the middle age group and adolescents ( *p* = 0.019). EDH is considered a life-threatening condition which can rapidly cause central brain herniation and subsequent cerebellar tonsillar herniation that is why early detection and surgical intervention are indicated. 8 In this review, all of the cases of EDH were sent back to the operating room and none of them were treated conservatively. In comparison to the other types of hemorrhage, majority of the EDH cases, 66.7% in this review, developed symptoms within 24 hours.

Twenty-six cases out of 109 cases in this study were identified to have SDH as complication of spine surgery (23.9%), 19.2% cases were less than 15 years of age. However, SDH was the type of ICH with the highest prevalence after spinal surgery among patients under 15 years as five out of eight patients were from the SDH group. Almost 54.0% of the cases were in the middle age group (15–65 years), and 26.9% were older than 65 years ( *p* = 0.024). SDH has been also reported following spinal procedures in another review with 25 cases reported following spinal anesthesia and 21 cases of subdural hematoma were reported after unintentional dural puncture following epidural anesthesia. 26 This suggests that SDH more commonly occurs after spinal procedures more than in spinal surgeries. There is still no study done for the comparison of ICH following spine surgery and spine procedure.

SDH has also been linked to the drop of intracranial pressure post–spine surgery which results from durotomy and CSF drainage or CSF leak. In this review, 46.1% of all SDH cases had a drain placed after the surgery and 19.2% reported CSF leak. In addition, some reports have established that loss of CSF after dural tear might be another important factor in the pathogenesis of ICH during or after spine surgery. This may lead to a headache caused by traction on the intracranial structures that are sensitive to pain. Headache post–spine surgery or post–lumbar puncture or spine anesthesia was noticed which can be a warning sign of SDH. A literature review done to identify if postspine procedure headache left untreated, it can lead to SDH, and it concluded that patient who develop post–dural puncture headache (PDPH), unrelieved by conservative measures, as well as the change of PDPH from postural to nonpostural, require careful follow-up for early diagnosis, and management of possible subdural hematoma. 25

In this review, it was found that out of 109 cases, 16.5% developed SAH after spinal surgery. SAH is a type of ICH that is more commonly associated with other types of hemorrhage such as cerebellar hemorrhage. Satake et al and Mallio et al wrote two case reports where SAH was associated with cerebellar hemorrhage. 5 27

Nineteen cases that were identified developed more than one type of hemorrhage, in comparison with those who develop only one type of hemorrhage, there was no effect on time of the symptoms, death was equal also in both groups. However, those who developed permanent complication following the ICH treatment were much higher among those who developed one type of hemorrhage. As 66.3% of one type of hemorrhage developed complication after treatment those compared with 36.8% who have more than one type of hemorrhage ( *p* = 0.034).

## Limitations

ICH post–spine surgery is a rare complication, and it generally tends to be underreported as not all case reports pass through the publication process. This indicates that the actual number of cases is under reported in the literature. However, the reported cases showed significant variability including the terminology use to report cases. Some data, such as comorbidities, amount of CSF leak, and the indication for spine surgery, were missing in most of the articles.

Heterogeneity of age, gender, type of spine surgery, and nonequal group of patients among the type of reported ICH raises a question on the accuracy of the analysis. Some patients who developed multiple types of hemorrhage were not excluded in our study which also might affect the analysis. Establishing a clear explanation for the real causes behind this rare complication was not applicable. The review did not include patients who developed hemorrhage after spinal procedures that did not need a surgical intervention where we could have analyzed variability of ICH type in procedures, such as lumbar puncture, and it only studied the prevalence of hemorrhage after spine surgery (i.e., the incidence was not studied). A true evaluation of the incidence and characteristics of these patients can only be done through a multicenter registry–based study.

## Conclusion

We presented a systematic review of 109 patients who developed ICH post–spine surgery identified from 79 case reports where cerebellar hemorrhage was found to be the most common type. ICH post–spine surgery is a rare complication and the real etiologies behind this complication are still unknown; however, this complication is thought to be secondary to durotomy and CSF leak. Reaching the correct diagnosis as early as possible is crucial to prevent aggravation of complications and to decrease the duration of hospitalization postoperatively. The incidence of this complication is still not well studied.
