# Supplementary material for: Intracranial Hemorrhage following Spinal Surgery: A Systematic Review of a Rare Complication
Source: Surg J (N Y). 2022 Mar 3;8(1):e98–e107. doi: 10.1055/s-0042-1743525 (PMC8894081; doi:10.1055/s-0042-1743525)
Supplement: Supplementary file 1 — Supplementary Material [file 10-1055-s-0042-1743525-s2100151.pdf]

Supplementary Material S1

No.	Article type	Main Author/year	No. of patients	Gender(s)	Spine surgery type	Intracranial hemorrhage type
1	Case report	Hofler/2018[1]	1	Female	Lumbar 2 to sacral 1 laminectomies and fusion	Bilateral curvilinear cerebellar hemispheric hemorrhages
2	Case report	Li/2012[2]	1	Female	Cervical degenerative cyst resection	Bilateral parietal EDH
3	Case report	Işik/2016[3]	1	Male	Lumbar laminectomy	Bilateral SDHs
4	Case report	Matsuhiro/2018[4]	1	Female	Posterior lumbar interbody fusion and lumbar laminectomy	SAH at the right cerebellopon-tine angle
5	Case report	Gul/2010[5]	1	Female	Lumbar laminectomies, lumbar discec-tomies and screw fixation of the L3-L5 pedicles, and posterior interbody fusion with allograft	Remote cerebellar hemorrhage
6	Case report	Yoo/2013[6]	1	Male	Laminotomy and intradural mass removal at L1-2	Bilateral cerebellar hemorrhage
7	Case report	Berry/2018[7]	1	Female	Anterior cervical discectomy, removal of cervical osteophytes, 8prosthetic disc replacement, bilateral cervical foraminoto-mies and a cervical spinal fusion	Bilateral basal ganglia hemorrhages with intraventricular extension and SAH
8	Case report	Mallio/2014[8]	1	Male	Decompressive lumbar laminectomy	Bilateral acute remote cere-bellar hemorrhage and bilat-eral supratentorial SAH
9	Case report	Burkhard/2000[9]	1	Male	Right L5-S1 diskectomy	Bilateral subacute SDHs
10	Case report	Satake/2000[10]	1	Male	Intramedullary tumor resection	Haemorrhage in the cere-bellum and supracerebellar subarachnoid layer
11	Case report	Konya/2005[11]	1	Female	Lumbar laminectomies, lumbar discectomy and screw fixation of the L3–L5 pedicles.	Bilateral cerebellar hemorrhage
12	Case report	Yang/2011[12]	1	Female	Lumbar spinal fusion	Cerebellar hemorrhage
13	Case report	Hashidate/2008[13]	1	Female	Thoracic laminectomy and extradural tumor resection	Bilateral cerebellar hemorrhage
14	Case report	Martínez-Lage/2015[14]	1	Male	Bilateral sacral laminectomy	Left convexity SDH
15	Case report	Farag/2005[15]	1	Female	Spinal re-exploration of L4-S1 and lateral fusion with iliac crest autograft	Bilateral cerebellar hemorrhage
16	Case report	Ozturk/2009[16]	1	Female	Rod placement for thoracolumbar scoliosis with posterior instrumenta-tion and fusion	Hemorrhages in the left cerebellar hemisphere, left temporal cortex and right putamen
17	Case report	Imajo/2016[17]	1	Male	Osteoplastic lumbar laminec-tomy and tumor resection	Left EDH
18	Case series	Yilmaz/2018[18]	8	1 Male<br>7 Females	Details are shown in Table 2	Details are shown in Table 2
19	Case series	Khalatbari/2012[19]	4	3 Males<br>1 Female	Details are shown in Table 2	Details are shown in Table 2
20	Case report	Habunaga/2011[20]	1	Male	Cervical open-door laminoplasty	Intracranial SDH
21	Case report	Lu/2002[21]	1	Female	Posterior laminectomy for L5 to S1 fusion with autologous iliac bone graft and stabilization with a variable screw placement system	Large right SDH
22	Case report	Nowak/2011[22]	1	Female	Thoracic transpedicular screw placement	Bilateral frontal SDHs
23	Case series	Pham/2015[23]	2	1 Male<br>1 Female	1- Male: C1 laminectomy with lateral mass resection and face-tectomy	1- Bilateral remote cerebellar hemorrhage<br>2- Left acute SDH

(*Continued*)

(*Continued*)

No.	Article type	Main Author/year	No. of patients	Gender(s)	Spine surgery type	Intracranial hemorrhage type
					2- Female: Thoracic costotransversectomy and thoracic spinal fusion	
24	Case report	Morandi/2001[24]	1	Male	C7–T1 laminectomy	Hemorrhage in the cerebellar hemispheres and vermis + hematoma in the left temporal region
25	Case series	Ma X/2013[25]	2	2 Females	1- Removal of subdural schwannoma 2- Posterior laminectomy through C2–C5, excision of an intraspinal mass and instrumental fusion from C2 to C5	1- Bilateral extradural cranial hematomas with mass eff ect at the posterior parietal region 2- Cerebellar hematoma and SAH
26	Case report	Brockmann/ 2005[26]	1	Female	Spinal fusion of L5–S1 using segmental instrumentation	Bilateral cerebellar hemispheres hemorrhage
27	Case report	Abel/2007[27]	1	Female	L5 laminectomy for transection of fatty filum terminale	Bilateral subdural fluid collections
28	Case report	Bowers/2011[28]	1	Female	S1–S2 laminectomies and resection of sacral chordoma	Supratentorial and cerebellar intraparenchymal hemorrhage
29	Case report	Elmaci/2000[29]	1	Male	- Decompressive laminectomy and resection of the thoracic disc - Resection of a T12-L1 disc by a transpedicular approach - Arthrodesis of two thoracic levels as well as one lumbar level using iliac crest bonegraft and pedicle screw fixation	Bilateral cerebellar hematomas
30	Case report	You/2012[30]	1	Male	L5-S1 discectomy, L3-L5 laminectomies, and screw fixation of the L3-L5 pedicle	Hemorrhages in the bilateral cerebellar hemispheres and the right temporal lobe
31	Case series	Friedman/2002[31]	2	1 Male 1 Female	1- Removal of the disc via a posterolateral transpedicular approach in the prone position 2- L3–S1 decompressive laminectomy and pedicle screw fixation	1- Cerebellar hemorrhage in the vermis and right hemisphere 2- Bilateral hemorrhage in the superior folia of the cerebellar vermis and the cerebellar hemispheres
32	Case report	Chadduk/1981[32]	1	Male	Cervical laminectomy	Hemorrhage in the left cerebellar hemisphere
33	Case report	Mikawa/1994[33]	1	Male	Left C1 hemilaminectomy and Brooks-type posterior hemifusion of C1 and C2	SAH in the posterior fossa with possible cerebellar hemorrhage
34	Case report	Thomas/2002[34]	1	Female	T11-L1 laminectomy and tumor excision	Bilateral hemorrhage in the temporal lobes and a larger hemorrhage in the right superior cerebellar hemisphere
35	Case report	Watanabe/2002[35]	1	Male	Total resection of thoracic spinal cord tumor with laminectomy	Right intracranial SDH
36	Case report	Andrews and Koci /1995[36]	1	Male	Harrington rod placement	Bilateral cerebellar hemorrhage
37	Case report	Nakazawa/2005[37]	1	Female	Laminectomy and durotomy at the level of C2 and resection of an intradural extramedullar tumor	Hemorrhage in the vermis and right cerebellar hemisphere
38	Case report	Sciubba/2005[38]	1	Female	Anterior L3 corpectomy with femoral allograft reconstruction and posterior L2–L4 pedicle screw fusion	Acute intracranial SDH with mass effect
39	Case report	Cornips/2007[39]	1	Female	T8-9 hernia removal	Left frontotemporal SDH, bilateral intraparenchymal hemorrhages and left cerebellar hemorrhage

 © 2022. The Author(s).

(*Continued*)

No.	Article type	Main Author/year	No. of patients	Gender(s)	Spine surgery type	Intracranial hemorrhage type
40	Case report	Enel/2009[40]	1	Female	Arthrodesis extension to a previous fusion	Bilateral cerebellar hemorrhage
41	Case report	Karaeminogullari/2005[41]	1	Female	Laminectomy, bilateral facetectomy at L3–L4 and L4–L5 and posterior instrumentation with pedicle screws from L2 to L5	Acute left cerebellar hematoma
42	Case series	Cevik/2009[42]	2	2 Females	1- L5 total laminectomy, L5 and S1 bilateral foraminectomy and screw fixation of the L4, L5 and S1 pedicles<br>2- L4 total laminectomy and L5 bilateral foraminectomy	1- SAH along the folia of both cerebellar hemispheres and in the vermis<br>2- Hemorrhage in the folia of both cerebellar hemispheres
43	Case report	Cavanilles-Walker/2013[43]	1	Female	Postero-lateral fusion and decompression from L2 to L5 with additional transforaminal interbody fusion at L3–4 and at L4–5	Bilateral cerebellar hemorrhage
44	Case series	Kaloostian/2013[44]	8	4 Males<br>4 Females	Details are shown in Table 2	Details are shown in Table 2
45	Case report	Miglis/2010[45]	1	Female	Significant CSF leakage after discectomy anf fusion surgery	Intracerebral hemorrhage in the left inferior temporal lobe
46	Case report	Halil Murat Sen/2017[46]	1	Male	L4–S1 posterior transpedicular fixation and L4–5 total laminectomy	bilateral cerebellar hemorrhage
47	Case report	Baran Yilmaz/2015[47]	1	Female	Lumbar laminectomy Posterior spinal instrumentation between s1 and s2	Reoperation, right intraprenchymal and subarachnoid
48	Case report	Gordan Grahovac/2011[48]	1	Female	Bilateral L3-L4 interlaminectomies and L3-L4 discectomy	extradural hematoma in the right parietal-occipital region
49	Case report	Muhammad Atif Naveed/2018[49]	1	Male	Elective spinal decompression revision surgery, with an extension of instrumented fusion from L2-L5.	acute haemorrhage in both cerebellar hemispheres,
50	Case report	Po-Hsien Huang/2013[50]	5	4 M<br>1 F	C3-c6 laminectomy<br>C3-c7 laminectomy<br>C3-c7 laminectomy,tumor resection<br>C2-5laminectomy<br>C2-5 laminectomy	2- Both CH<br>2- Both CH,with hydrocephalous<br>Left CH
51	Case report	Estela Val-Jordán/2017[51]	1	Female	L4-L5 lumbar arthrodesis	Right CH,hydropcephaly, SAH,SH
52	Case report	Yizhar Floman/2015[52]	3	2 F<br>1 M	Decompression laminectomy<br>L4-5 decompression<br>Decompression	Hemorrhage in the left posterior fossa<br>Bilateral ch with occlusion of ventricle<br>Hematoma in cerebellum
53	Case report	Masazumi Suzuki/2015[53]	1	Female	Tumor resection, laminectomy T2-T4, facetectomy T2-T3 and T3-T4, T1-T5 pedicle screw fixation was performed	Acute cerebellar hemorrhage in the superior folia of the cerebellar hemispheres
54	Case report	Shotaro Watanabe/2015[54]	1	Male	Removal of the tumor and posterolateral fusion	Hemorrhage cerebellum and the temporal lobe
55	Retrospective	Toshimi Aizawa/2018[55]	1 full report<br>2 not completed	2 females<br>1 male	Tumor resection, laminectomy T10-S1	Intracranial hemorrhage in the right cerebellar hemisphere, tentorium cerebelli, and the left temporal lobe
56	Case report	Ralf G. Hempelmann/2012[56]	3	Female	Laminectomy and posterior instrumentation L2–4<br>Spinal decompression by laminectomy of L3 and intervertebral fusion<br>Tumor hemilaminectomy of Th 1–4	parieto-occipital cerebral haemorrhages as well as minor cerebellar bleedings on both sides<br>Hematoma in cerebellum<br>cerebellar haemorrhage in the right hemisphere
57	Case report	Javier Fernandez-Jara/2011[57]	1	Female		

(*Continued*)

(*Continued*)

No.	Article type	Main Author/year	No. of patients	Gender(s)	Spine surgery type	Intracranial hemorrhage type
					L5-S1 laminectomy with trans-pedicular fixation	subarachnoid bleeding, intraparenchymal bleeding and vasogenic edema
58	Case report	Peter Khong/2009[58]	1	Female	L5–S1 laminot- omy, foraminot-omy and rhizolysis	cerebellar haemorrhage
59	Case report	Gokhan Bozkurt/2016[59]	1	Female	Decompressive laminectomy	sylvian-periinsular subarach-noid hemorrhage and pneumo- cephalus in the right cerebral hemisphere
60	Case report	Ufuk Utku/2013[60]	1	Male	Laminectomy by exposing T12-L5 and had pedicle screws placed	left cerebellar hemi- sphere subdural hematoma at right frontotemporal
61	Case report	Rohan Bhimani/2017[61]	1	Male	Detethering at the L2 level with pedicular screw fixation from T3 to L2 with bone grafting with right costoplasty from the 3rd to the 6th ribs	subdural hemorrhage
62	Case report	Gilberto Ka Kit Leung/2014[62]	1	Female	Laminectomy for tumour excision	signifi- cant brain swelling and multiple ICHs
63	Case report	Kirişoğlu M Ü/2015[63]	1	Female	T5 and 6 laminectomy Tumor resection	right parietooccipital intra-cerebral hemorrhage
64	Case report	Tadatsugu Morimoto/2014[64]	1	Male	Occipitocervical fusion and C1 pos- terior arch resection	SDH and CH
65	Case report	Zakaria AF/2019[65]	1	Female	Cyst Excision and decompres-sion Laminectomy s1	Right SDH
66	Case report	Richard A Wawrose/2019[66]	1	Female	L3–L4 lateral lumbar interbody fusion (LLIF), followed by L3–L4 revision laminectomy and revi-sion in- strumented PSF from L2–L5	right-sided chronic SDH
67	Case report	Yoichi Morofuji/2009[67]	1	Male	T9-T10 laminectomy and resec-tion of the OLF	Bilateral cerebellar hemor-rhages facing the tentorium and obstructive hydrocephalus
68	Case report	Yuichi Takahashi/ 2012[68]	1	Male	Cervical laminoplasty (C3–7) for cervical spon- dylotic myelopa-thy without intraoperative liquorrhea.	Cerebellar hemorrhage, acute subdural hemorrhage, subarachnoid hemorrhage, supratentorial in- traparen-chymal hemorrhage, and pneumocephalus
69	Case report	Johan Pallud/2009[69]	1	Female	L5-S1 laminectomy and fusion with pedicle screws in prone position using a posterior ap-proach in an outside institution	External ventriculostomy Decompressive craniectomy with a large duraplasty was then performed
70	Case report	Justin M. Haller/2016[70]	1	Female	L3–L4 decompressive laminec-tomy and posterior instru-mented spinal fusion.	Bilateral cerebellar paren-chymal hemorrhage with mass effect and inferior dis-placement of the cerebellar tonsils
71	Case report	Youn Young Jung/2010[71]	1	Male	Posterior lumbar interbody fusion	Acute sub- dural hematomas at the frontoparietal area, bilaterally
72	Case report	Julio A. Chalela,/2006[72]	1	Male	Laminectomy at the above-mentioned levels (l3-l5)	Hem- orrhage in the superior aspects of the cerebellum and acute obstructive hydrocephalus
73	Case report	Mehdi Sasani/2009[73]	1	Female	T12,L1,L2 laminoplasty	Subacute bilateral intrapa-ren- chymal cerebellar hemorrhage
74	Case report	Tarkan Calisaneller/2007[74]	1	Female	Decompressive L5-laminectomy with L4-L5- S1 posterior trans-pedicular screw-rod fixations was performed	bilateral haemorrhages in the cerebellar hemispheres

(*Continued*)

No.	Article type	Main Author/year	No. of patients	Gender(s)	Spine surgery type	Intracranial hemorrhage type
75	Case report	Do Keun Kim/2010[75]	1	Female	Spinal fusion	Right side sided ICH with edema, ventricular hemor- rhage, and sub- arachnoid hemorrhage (SAH) with mass effect<br>Coma MRI<br>low signal change at both hemisphere (right > left) with moderate hy- droce- phalus (hydrocephalic index = 45%)
76	Case report	Alexandra D/2009[76]	1	Female	S1 radiculopathy. L5–S1 herni- ated nucleus pulposis	right chronic frontoparietal SDH with 8-mm midline shift and a lumbar pseudomeningocele
77	Case report	Ji Yong Kim/2015[77]	1	Female	Laminectomy at L4-L5 Pedicle-screw fixation were performed	re- vealed a cerebellar hem- orrhage in the right cerebel- lar hemi- sphere and vermis
78	Case report	Taek Kyun Nam/ 2009[78]	1	Male	Partial hemilaminectomy and discectomy at local spine clinic	subo- ccipital craniectomy, hematoma removal, duro- plasty, and CSF diversion via extraventricular drainage from right lateral ventricle to prevent hydrocephalus
79	Case report	Hironobu Sakaura /2006[79]	1	Male		

References

1 Hofler RC, Wemhoff MP, Johans SJ, Nockels RP. Cerebellar Hemorrhage Following an Uncomplicated Lumbar Spine Surgery: Case Report. J Stroke Cerebrovasc Dis 2019;28(07):e104–e105

2 Li Z-J, Sun P, Dou YH, et al. Bilateral supratentorial epidural hematomas: a rare complication in adolescent spine surgery. Neurol Med Chir (Tokyo) 2012;52(09):646–648. Doi: 10.2176/nmc.52.646

3 Işik S, Yilmaz B, Ekşi MŞ, et al. Delayed Onset Intracranial Subdural Hematoma Following Spinal Surgery. J Craniofac Surg 2016;27(04):e370–e373

4 Matsuhiro J, Kariyazono R, Mizutani K, Hinotsume A, Tsuchiya M. A case report of seizure during emergence from general anesthesia after lumbar spinal surgery-common cases can develop potentially life-threatening adverse intracranial events. JA Clin Rep 2018;4(01):42

5 Gul S, Kalayci M, Acikgoz B. Cerebellar hemorrhage: as a rare complication of spinal surgery. Turk Neurosurg 2009

6 Yoo JC, Choi JJ, Lee DW, Lee S. Remote cerebellar hemorrhage after intradural disc surgery. J Korean Neurosurg Soc 2013;53(02):118–120

7 Berry B, Ghannam M, Bell C, et al. Basal ganglia hemorrhage in a case report following spinal surgery. BMC Neurol 2018;18(01):204. Doi: 10.1186/s12883-018-1218-x

8 Mallio CA, Sarà M, Pistoia ML, et al. Bilateral remote cerebellar haemorrhage after spinal surgery: a case study and review of the literature. Brain Inj 2014;28(09):1216–1222. Doi: 10.3109/02699052.2014.919524

9 Burkhard PR, Duff JM. Bilateral subdural hematomas following routine lumbar diskectomy. Headache 2000;40(06):480–482

10 Satake K, Matsuyama Y, Iwata H, Sato K, Kawakami N. Cerebellar haemorrhage complicating resection of a cervical intramedullary tumour. Spinal Cord 2000;38(08):504. Doi: 10.1038/sj.sc.3101020

11 Konya D, Ozgen S, Pamir MN. Cerebellar hemorrhage after spinal surgery: case report and review of the literature. Eur Spine J 2006;15(01):95–99. Doi: 10.1007/s00586-005-0987-2

12 Yang KH, Han JU, Jung JK, Lee DI, Hwang SI, Lim HK. Cerebellar hemorrhage after spine fixation misdiagnosed as a complication of narcotics use -A case report-. Korean J Anesthesiol 2011;60(01):54–56

13 Hashidate H, Kamimura M, Nakagawa H, Takahara K, Uchiyama S, Kato H. Cerebellar hemorrhage after spine surgery. J Orthop Sci 2008;13(02):150–154

14 Martínez-Lage JF, López-Guerrero AL, Piqueras C, Almagro MJ, Gilabert A. Intracranial hemorrhage following surgery for occult spinal dysraphism: a case-based update. Childs Nerv Syst 2015;31(06):837–842

15 Farag E, Abdou A, Riad I, Borsellino SR, Schubert A. Cerebellar hemorrhage caused by cerebrospinal fluid leak after spine surgery. Anesth Analg 2005;100(02):545–546

16 Ozturk E, Kantarci M, Karaman K, Basekim CC, Kizilkaya E. Diffuse pneumocephalus associated with infratentorial and supratentorial hemorrhages as a complication of spinal surgery. Acta Radiol 2006;47(05):497–500

17 Imajo Y, Kanchiku T, Suzuki H, et al. Intracranial epidural hemorrhage during lumbar spinal surgery. Spinal Cord Ser Cases 2016;2(01):15040

18 Yilmaz B, Toktas Z, Konya D, Hasanov T. INTRACRANIAL COMPLICATIONS OF LUMBAR SPINAL SURGERY. The Journal of Turkish Spinal Surgery 2018;29(03):165–172

19 Khalatbari MR, Khalatbari I, Moharamzad Y. Intracranial hemorrhage following lumbar spine surgery. Eur Spine J 2012;21(10):2091–2096. Doi: 10.1007/s00586-012-2187-1

20 Habunaga H, Nakamura H. Intracranial subdural hematoma as a cause of postoperative delirium and headache in cervical laminoplasty: A case report and review of the literature. SAS J 2011;5(01):1–3

21 Lu CH, Ho ST, Kong SS, Cherng CH, Wong CS. Intracranial subdural hematoma after unintended durotomy during spine surgery. Can J Anaesth 2002;49(01):100–102

22 Nowak R, Maliszewski M, Krawczyk L. Intracranial subdural hematoma and pneumocephalus after spinal instrumentation

of myelodysplastic scoliosis. J Pediatr Orthop B 2011;20(01): 41–45
23 Pham MH, Tuchman A, Platt A, Hsieh PC. Intracranial complications associated with spinal surgery. Eur Spine J 2016;25(03): 888–894. Doi: 10.1007/s00586-015-4241-2
24 Morandi X, Riffaud L, Carsin-Nicol B, Guegan Y. Intracerebral hemorrhage complicating cervical “hourglass” schwannoma removal. Case report. J Neurosurg 2001;94(1, Suppl)150–153
25 Ma X, Zhang Y, Wang T, et al. Acute intracranial hematoma formation following excision of a cervical subdural tumor: a report of two cases and literature review. Br J Neurosurg 2014; 28(01):125–130
26 Brockmann MA, Nowak G, Reusche E, Russlies M, Petersen D. Zebra sign: cerebellar bleeding pattern characteristic of cerebrospinal fluid loss. Case report. J Neurosurg 2005;102(06): 1159–1162
27 Abel TJ, Chowdhary A, Gabikian P, Ojemann JG, Ellenbogen RG, Avellino AM. Spontaneous subdural fluid collections following transection of a fatty filum terminale: case report and review of the literature. Pediatr Neurosurg 2007;43(06):507–511
28 Bowers CA, Taussky P, Duhon BS, Schmidt MH. Multiple supra- and infratentorial intraparenchymal hemorrhages presenting with seizure after massive sacral cerebrospinal fluid drainage. Spine 2011; 36(04):E288–E291. Doi: 10.1097/BRS.0b013e3181f9b10f
29 Elmaci I, Ain MC, Wright MJ, et al. Perioperative intracranial hemorrhage in achondroplasia: a case report. J Neurosurg Anesthesiol 2000;12(03):217–220
30 You SH, Son KR, Lee NJ, Suh JK. Remote cerebral and cerebellar hemorrhage after massive cerebrospinal fluid leakage. J Korean Neurosurg Soc 2012;51(04):240–243
31 Friedman JA, Ecker RD, Piepgras DG, Duke DA. Cerebellar hemorrhage after spinal surgery: report of two cases and literature review. Neurosurgery 2002;50(06):1361–1363, discussion 1363–1364. Doi: 10.1097/00006123-200206000-00030
32 Chadduck WM. Cerebellar hemorrhage complicating cervical laminectomy. Neurosurgery 1981;9(02):185–189. Doi: 10.1227/00006123-198108000-00016
33 Mikawa Y, Watanabe R, Hino Y, Ishii R, Hirano K. Cerebellar hemorrhage complicating cervical durotomy and revision C1-C2 fusion. Spine 1994;19(10, Supplement)1169–1171
34 Thomas G, Jayaram H, Cudlip S, Powell M. Supratentorial and infratentorial intraparenchymal hemorrhage secondary to intracranial CSF hypotension following spinal surgery. Spine 2002;27 (18):E410–E412
35 Watanabe A, Takai H, Ogino S, Ohki T, Ohki I. Intracranial subdural hematoma after resection of a thoracic spinal cord tumor. J Spinal Disord Tech 2002;15(06):533–536
36 Andrews RT, Koci TM. Cerebellar herniation and infarction as a complication of an occult postoperative lumbar dural defect. AJNR Am J Neuroradiol 1995;16(06):1312–1315
37 Nakazawa K, Yamamoto M, Murai K, Ishikawa S, Uchida T, Makita K. Delayed emergence from anesthesia resulting from cerebellar hemorrhage during cervical spine surgery. Anesth Analg 2005; 100(05):1470–1471
38 Sciubba DM, Kretzer RM, Wang PP. Acute intracranial subdural hematoma following a lumbar CSF leak caused by spine surgery. Spine 2005;30(24):E730–E732. Doi: 10.21037/atm.2019.01.04
39 Cornips EM, Staals J, Stavast A, Rijkers K, van Oostenbrugge RJ. Fatal cerebral and cerebellar hemorrhagic infarction after thoracoscopic microdiscectomy. Case report. J Neurosurg Spine 2007; 6(03):276–279
40 Enel D, Blamoutier A, Bacon P, Gentili ME. Spine surgery associated with fatal cerebellar haemorrhage. Eur J Anaesthesiol 2009;26 (10):891–892
41 Karaeminogullari O, Atalay B, Sahin O, et al. Remote cerebellar hemorrhage after a spinal surgery complicated by dural tear: case report and literature review. Neurosurgery 2005;57(1, Suppl)E215–, discussion E215. Doi: 10.1227/01.neu.0000163688.17385.9b
42 Cevik B, Kirbas I, Cakir B, Akin K, Teksam M. Remote cerebellar hemorrhage after lumbar spinal surgery. Eur J Radiol 2009;70 (01):7–9
43 Cavanilles-Walker JM, Tomasi SO, Sgier F, Kröber M. Remote cerebellar haemorrhage after lumbar spine surgery: case report. Arch Orthop Trauma Surg 2013;133(12):1645–1648. Doi: 10.1007/s00402-013-1867-6
44 Kaloostian PE, Kim JE, Bydon A, et al. Intracranial hemorrhage after spine surgery. J Neurosurg Spine 2013;19(03):370–380. Doi: 10.3171/2013.6.SPINE12863
45 Miglis MG, Levine DN. Intracranial venous thrombosis after placement of a lumbar drain. Neurocrit Care 2010;12(01):83–87
46 Sen HM, Guven M, Aras AB, Cosar M. Remote cerebellar hemorrhage presenting with cerebellar mutism after spinal surgery: An unusual case report. J Korean Neurosurg Soc 2017;60(03): 367–370
47 Yılmaz B, Işık S, Ekşi MŞ, et al. Multiple hemorrhages in brain after spine surgery supra- and infra-tentorial components together. J Craniovertebr Junction Spine 2015;6(04):223–226
48 Grahovac G, Vilendecic M, Chudy D, Srdoc D, Skrlin J. Nightmare complication after lumbar disc surgery: cranial nontraumatic acute epidural hematoma. Spine 2011;36(26):E1761–E1764
49 Naveed MA, Mangla R, Idrees H, Mehta RI. Remote Cerebellar Haemorrhage: A Potential Iatrogenic Complication of Spinal Surgery. Case Rep Neurol Med 2018;2018:5870584
50 Huang PH, Wu JC, Cheng H, Shih YH, Huang WC. Remote cerebellar hemorrhage after cervical spinal surgery. J Chin Med Assoc 2013; 76(10):593–598
51 Val-Jordán E, Seral-Moral P, Novo-González B. Remote cerebellar hemorrhage caused by undetected dural tear after lumbar spinal surgery. Rev Esp Cir Ortop Traumatol (Engl Ed) 2018;62(03): 228–230(English Edition)
52 Floman Y, Millgram MA, Ashkenazi E, Rand N. Remote cerebellar hemorrhage complicating unintended durotomy in lumbar spine surgery. Int J Spine Surg 2015;9:29
53 Suzuki M, Kobayashi T, Miyakoshi N, Abe E, Abe T, Shimada Y. Remote cerebellar hemorrhage following thoracic spinal surgery of an intradural extramedullary tumor: a case report. J Med Case Reports 2015;9(01):68
54 Watanabe S, Ohtori S, Orita S, et al. Remote Hemorrhage in the Cerebellum and Temporal Lobe after Lumbar Spine Surgery. Case Rep Orthop 2015;2015:972798
55 Aizawa T, Ozawa H, Ashina Y, Hashimoto K, Kanno H, Eto T. Remote Intracranial Hemorrhage after Spinal Surgery: Possible Etiology and Incidence. Clinics in Surgery. 2018;•••:3
56 Aizawa T, Ozawa H, Ashina Y, Hashimoto K, Kanno H, Eto T, et al. Remote Intracranial Hemorrhage after Spinal Surgery: Possible Etiology and Incidence. Clin Surg 2018;3:2058
57 Fernandez-Jara J, Jorge-Blanco A, Carro-Martinez AI, Ferreiro-Argüelles C, Fernandez-Gallardo JM, Romero-Coronado J. Remote cerebellar hemorrhage after lumbar spinal surgery. Emerg Radiol 2011;18(02):177–180
58 Khong P, Jerry Day M. Spontaneous cerebellar haemorrhage following lumbar fusion. J Clin Neurosci 2009;16(12): 1673–1675
59 Bozkurt G, Yaman ME, Yaman ME. Subarachnoid Hemorrhage Presenting with Seizure due to Cerebrospinal Fluid Leakage after Spinal Surgery. J Korean Neurosurg Soc 2016;59(01):62–64. Doi: 10.3340/jkns.2016.59.1.62
60 Utku U, Güler S, Yalnız E, Unlü E Subdural and cerebellar hematomas which developed after spinal surgery: a case report and review of the literature. Case Rep Neurol Med 2013;2013:431261
61 Bhimani R, Bhimani F, Singh P. Subdural Hemorrhage after Scoliosis and Detethering of Cord Surgery. Case Rep Med 2018; 2018:5061898
62 Leung GK, Chan JP. Supratentorial Intraparenchymal Haemorrhages during Spine Surgery. J Korean Neurosurg Soc 2014;55 (02):103–105

63 Kirişoğlu MÜ, Ösün A, Atay B, Samancıoğlu A. Supratentorial Intraparenchymal Hemorrhage after Spinal Meningioma Surgery. J Biomed Sci 2015;04(02):
64 Morimoto T, Shiraki M, Otani K, Sonohata M, Mawatari M. Supratentorial subdural hemorrhage of a previous head injury and cerebellar hemorrhage after cervical spinal surgery: a case report and review of the literature. Spine 2014;39(12):E743–E747
65 Zakaria AF, Tsuji M. Intracranial Subdural Hematoma after Lumbar Spine Surgery: A Case Report. Malays Orthop J 2019;13(03):85–87
66 Wawrose RA, Dombrowski ME, Crasto JA, Shaw JD, Lee JY. Chronic Subdural Hematoma as a Complication of Cerebrospinal Fluid Leak During Revision Lumbar Spine Surgery: A Case Report and Review of the Literature. HSS J 2020;16(Suppl 2):482–484
67 Morofuji Y, Tsunoda K, Takeshita T, et al. Remote cerebellar hemorrhage following thoracic spinal surgery. Neurol Med Chir (Tokyo) 2009;49(03):117–119
68 Takahashi Y, Nishida K, Ogawa K, et al. Multiple intracranial hemorrhages after cervical spinal surgery. Neurol Med Chir (Tokyo) 2012;52(09):643–645
69 Pallud J, Belaïd H, Aldea S. Successfull management of a life threatening cerebellar haemorrhage following spine surgery - a case report -. Asian Spine J 2009;3(01):32–34
70 Haller JM, Calvert G, Spiker WR, Brodke DS, Lawrence BD. Remote Cerebellar Hemorrhage after Revision Lumbar Spine Surgery. Global Spine J 2015;5(06):535–537
71 Jung YY, Ju CI, Kim SW. Bilateral Subdural Hematoma due to an Unnoticed Dural Tear during Spine Surgery. J Korean Neurosurg Soc 2010;47(04):316–318
72 Chalela JA, Monroe T, Kelley M, et al. Cerebellar hemorrhage caused by remote neurological surgery. Neurocrit Care 2006;5(01):30–34
73 Sasani M, Sasani H, Ozer AF. Bilateral late remote cerebellar hemorrhage as a complication of a lumbo-peritoneal shunt applied after spinal arteriovenous malformation surgery. J Spinal Cord Med 2010;33(01):77–79
74 Calisaneller T, Yilmaz C, Ozger O, Caner H, Altinors N. Remote cerebellar haemorrhage after spinal surgery. Can J Neurol Sci 2007;34(04):483–484
75 Kim D, Park C, Yoon S, Hyun D. Remote Cerebellar Hemorrhage after Spinal Surgery. Journal of Korean Neurotraumatology Society. 2010;6(02):162
76 Beier AD, Soo TM, Claybrooks R. Subdural hematoma after microdiscectomy: a case report and review of the literature. Spine J 2009;9(10):e9–e12
77 Kim JY, Kim K, Yoon SH. Remote Cerebellar Hemorrhage after Surgery for Degenerative Lumbar Spine Disease: A Case Report. Korean J Neurotrauma 2015;11(02):201–204
78 Nam TK, Park SW, Min BK, Hwang SN, Hwang SN. Remote cerebellar hemorrhage after lumbar spinal surgery. J Korean Neurosurg Soc 2009;46(05):501–504. Doi: 10.3340/jkns.2009.46.5.501
79 Sakaura H, Hosono N, Mukai Y, Ishii T, Yoshikawa H. Multiple cerebellar hemorrhagic infarctions following surgery for a huge atlantoaxial neurinoma. Spine J 2006;6(01):86–89

 © 2022. The Author(s).
